# The environmental consequences of climate-driven agricultural frontiers

**DOI:** 10.1371/journal.pone.0228305

**Published:** 2020-02-12

**Authors:** Lee Hannah, Patrick R. Roehrdanz, Krishna Bahadur K. C., Evan D. G. Fraser, Camila I. Donatti, Leonardo Saenz, Timothy Max Wright, Robert J. Hijmans, Mark Mulligan, Aaron Berg, Arnout van Soesbergen

**Affiliations:** 1 The Betty and Gordon Moore Center for Science, Conservation International, Arlington, Virginia, United States of America; 2 Department of Geography, Environment and Geomatics, University of Guelph, Guelph, Ontario, Canada; 3 Arrell Food Institute and the Department of Geography, Environment and Geomatics, University of Guelph, Guelph, Ontario, Canada; 4 Michigan Technological University, Houghton, Michigan, United States of America; 5 Department of Environmental Science and Policy, University of California Davis, Davis, California, United States of America; 6 Department of Geography, Kings College London, London, United Kingdom; 7 UN Environment World Conservation Monitoring Centre, Cambridge, United Kingdom; Universidade de Vigo, SPAIN

## Abstract

Growing conditions for crops such as coffee and wine grapes are shifting to track climate change. Research on these crop responses has focused principally on impacts to food production impacts, but evidence is emerging that they may have serious environmental consequences as well. Recent research has documented potential environmental impacts of shifting cropping patterns, including impacts on water, wildlife, pollinator interaction, carbon storage and nature conservation, on national to global scales. Multiple crops will be moving in response to shifting climatic suitability, and the cumulative environmental effects of these multi-crop shifts at global scales is not known. Here we model for the first time multiple major global commodity crop suitability changes due to climate change, to estimate the impacts of new crop suitability on water, biodiversity and carbon storage. Areas that become newly suitable for one or more crops are Climate-driven Agricultural Frontiers. These frontiers cover an area equivalent to over 30% of the current agricultural land on the planet and have major potential impacts on biodiversity in tropical mountains, on water resources downstream and on carbon storage in high latitude lands. Frontier soils contain up to 177 Gt of C, which might be subject to release, which is the equivalent of over a century of current United States CO_2_ emissions. Watersheds serving over 1.8 billion people would be impacted by the cultivation of the climate-driven frontiers. Frontiers intersect 19 global biodiversity hotspots and the habitat of 20% of all global restricted range birds. Sound planning and management of climate-driven agricultural frontiers can therefore help reduce globally significant impacts on people, ecosystems and the climate system.

## Introduction

One of the key challenges facing the 21^st^-century is producing enough food for the world’s growing population while not undermining the ecosystems on which we depend for life [1]. In particular, it is argued that the world needs to produce 70% more food by 2050 and if we fail to achieve this then there will be major social and economic impacts [2–4]. Proposed solutions to the global challenge of sustainably feeding the world’s population include consumer shifts towards more plant-based diets, reducing food waste, new technologies to boost yields, applying existing technology to close yield gaps, and expanding the amount of land currently under cultivation. Here we assess the extent to which climate change may create new opportunities to cultivate land in regions not currently cultivated as well as assessing the environmental implications of developing these so-called “agricultural frontiers.” In particular, climate change may stimulate large-scale geographic shifts in lands suitable for agricultural production, including the expansion of cultivation at the thermal and precipitation limits of crop tolerance [5–10]. Already climate change is creating new opportunities for farming to expansion in higher altitudes and latitudes that will enjoy longer growing seasons [7, 11–12]. Unfortunately, the environmental consequences of these climate-driven agricultural frontiers are not fully appreciated [11–15].

Balancing cropland expansion, to feed the world’s growing population, with the protection of land to conserve biodiversity and ecosystem services is a major global challenge [2]. Over the past 50 years, increases in food production have been dominated by growth in yields. Now, increasingly we are seeing expansion of agricultural lands through large-scale clearing [16]. 27% of global deforestation is directly attributable to large-scale clearing for commodity production, predominantly in the tropics [17].

In this paper, we define agricultural frontiers as areas not currently suitable for any major global commodity but that become suitable in the future due to climate change. While these areas present an opportunity for agricultural expansion, concern lays in the possibility of environmental degradation that may accompany development of frontiers. The expansion of agriculture into newly suitable regions may lead to environmental impacts not experienced under previous land uses, including impacts on biodiversity, water services and carbon storage [18]. Therefore, policies to optimize food production, biodiversity and ecosystem services under climate change are needed, especially since many past and present government policies have, intentionally and unintentionally, favored agricultural expansion.

While global crop models have repeatedly identified areas of new agricultural suitability that open due to climate change, analyses to date, for example using intersectoral impact models and earth system models, have not fully elaborated the environmental impacts specific to those areas [9, 19]. One reason is that global models often combine many sectors and varying assumptions [20] and are run at a relatively coarse resolution that may not match the scale of environmental qualities of concern (e.g. watersheds, species ranges). In this study, we use simple but high-resolution crop suitability models to document possible water, carbon and biodiversity impacts associated with the potential cultivation of areas becoming suitable for agriculture for the first time. Through this analysis we aim to improve understanding of the potential implications of the expansion of agriculture into agricultural frontiers, to inform policies that balance optimized food production with the importance of biodiversity and ecosystem services.

## Materials and methods

### Climate data

We identify climate-driven agricultural frontiers by using projections of 17 global climate models (GCM) for two levels of radiative forcing (Representative Concentration Pathways; RCPs 4.5 and 8.5) (S1 Table). S1 Fig presents a flowchart showing the details of GCMs and other data used and their analysis.

Current and future monthly climate grids were obtained from WorldClim Global Climate Data (www.worldclim.org). All data obtained was downscaled to 30 arc-second (approx. 1km) horizontal resolution following the methods of Hijmans et al. 2005 [21]. Variables available for download at this resolution and used in the simulations reported here were mean daily maximum temperature of each month (Tmax), mean daily minimum temperature of each month (Tmin), mean total precipitation of each month (Precip) and a suite of 19 bioclimatic variables [S2 Table]. Current climate represents the mean monthly climate over the period 1950–2000 whereas future climate projections cover 20-year averages over the periods 2040–2060 and 2060–2080.

### Crop suitability models

Climatic suitability for twelve globally important crops is determined using three discrete modeling methods 1) EcoCrop; 2) Maxent; 3) frequency of daily critical maximum and minimum temperatures. The list of crops is listed in Table 1 and parameters used is depicted in S3 Table.

**Table 1:** List of crops modeled.

Crop Name	Species
Corn	*Zea mays*
Sugar	*Saccharum officinarum*
Oil Palm	*Elaeis guineensis*
Cassava	*Manihot esculenta*
Peanuts	*Arachis hypogaea*
Cotton	*Gossypium hirsutum*
Millet	*Pennisetum glaucum*
Sorghum	*Sorghum halepense*
Rice	*Oryza sativa*
Potato	*Solanum tuberosum*
Wheat	*Triticum aestivum*
Soy	*Glycine max*

EcoCrop is a generalized physiological model of crop suitability based on known ranges of optimal temperature and precipitation as well as climatic limits where production of that crop would be impossible [22, 23]. Parameters for optimal temperature and precipitation are available from the EcoCrop database maintained by the FAO [24].

Maximum entropy (Maxent) is widely used in modeling species distributions under climate change and increasingly applied to domesticated crop species [25]. Crop occurrence points were generated from spatially weighted random points representing the agreement of four discrete gridded crop databases (S4 Table). One thousand points were generated for each crop, with a greater percentage of the distribution given where there is greater agreement among the four databases. Ten variables were selected by hierarchical clustering to create the Maxent model for each crop. For each crop, all 19 bioclimatic variables were sampled at the crop observation points. Variables were standardized then partitioned into clusters using hclust() base R function. The resulting cluster dendrogram was partitioned into 10 clusters using cutree() base R function where k = 10. The first variable was selected from each of the resulting 10 groups. See S2 and S5 Tables for a description of the bioclimatic variables used. For all models, 30% of the occurrence points were reserved to test and validate the model. Model performance (AUC), logistic threshold and top four most important variables for each crop are shown in S6 Table.

Critical maximum and minimum temperatures critical to crop success [26] were modeled with gridded global daily observations for maximum and minimum air temperature were obtained from the NOAA Earth System Research Laboratory Twentieth Century Reanalysis Version 2: 4-times daily and daily average monolevel dataset [27] at 2° x 2° resolution.

Global daily observation grids (2° x 2°) were summarized by month to create a count of days where temperature exceeded each integer degree in the range -50C to +50C. Temperatures were rounded to the nearest degree C and summarized in one-degree intervals. Counts of temperature exceedances were generated over a 20-year period (1980–2000) and resampled to 30 arc seconds (see S7 Table). Binary suitable vs. not-suitable grids were then created by thresholding the critical temperature counts at 20% of all days within the relevant month over the 20-year period using a cropping calendar dataset [28].

### Agricultural frontier ensemble

Suitability for each crop was determined for current climate and in each of 34 future scenarios (17 GCM, 2 radiative forcing) as the agreement of the three suitability methods (EcoCrop, Maxent, Critical temperatures). Frontiers are defined as areas that transition from zero crops suitable in current climate to one or more crops suitable in the future climate scenario. Additionally, we mapped frontiers that transition from one crop suitable in current climate to two or more crops suitable in the future climate scenario. Validation statistics as measured by Dice-Sorenson spatial congruence for all crop models are shown in S8 Table.

### Water quality impacts

The analysis was implemented in a hydrological model, WaterWorld version 2, globally at 10km spatial resolution [29]. To calculate the population within the new agricultural areas, who may experience water quality declines, we sum the Landscan global population dataset 2011 [30] over the area of new agriculture. The WaterWorld *hydrological footprint (HF)* [29–31] is a measure of the influence of an area on downstream water and is calculated by cumulating a global water balance calculated by the WaterWorld model [31], along a HydroSHEDS [32] based flow network. The hydrological footprint at a point along this network is defined as the contribution of an upstream area (such as a new agricultural area) to flow of water at this point. We calculate the Agricultural Water Quality (AWQ) index as the land area affected by hydrological footprint >0% and >50% as proxies for all contamination and for significant contamination respectively. Further, we calculate the population affected by this hydrological footprint as the sum of Landscan 2011 [30] people in pixels with a hydrological footprint of the new agricultural areas >0% and >50%. We use a global database of 38,000 dams [33] to identify which lie on rivers with a hydrological footprint>0 as a surrogate for agricultural, hydropower and urban water supplies potentially affected by this new agricultural land. Footprints are calculated for the current distribution of cropland fractional cover [34], the current distribution of land suitable for cropland and each ensemble member of the frontiers. We used a “difference method” where the number of people affected is a function of the agricultural water footprint of *current* farming systems, minus the agricultural water footprint of *future* farming practices. We use this difference to calculate the fraction of water, at any point in the hydrological network, that fell as rain on particular land uses upstream and thus is likely to be contaminated by new cropland.

### Soil organic carbon impacts

To account for soil organic carbon stocks in agricultural frontiers, we obtained a gridded global dataset of estimated soil organic carbon (tonnes ha^-1^) in the top 100 cm [35]. Individual projections of agricultural frontiers were resampled to match the native resolution of the dataset and used to extract the soil carbon values for areas within the frontier. Total tonnage of soil carbon was then summed for each resulting grid. To assess the total emissions resulting from conversion from natural land cover to agriculture, we employed land cover-specific emissions rates based on several meta analyses [36*–*38] as well as IPCC best practices guidelines for carbon accounting under land use change [39]. To account for differential emissions assumptions by land class, the areas in agricultural frontiers were categorized according to existing land cover in the global land cover facility (GLCF) dataset [40*–*41].

### Biodiversity impacts

Biodiversity hotspots [42], endemic bird areas (EBA) [43], and Key Biodiversity Areas (KBA) [44*–*45] were assessed for overlap with agricultural frontiers, as were present and future ranges of global restricted-range bird species. Hotspots, EBA and KBA polygons were converted to raster. Occurrence data and range maps for global restricted range bird species were obtained from Birdlife International [46]. Only species whose observed area of occupancy was within 100 km of a frontier and with >10 occurrence points were modeled. Maxent models were generated for all selected species for baseline climate and in all future climate scenarios for the 17 GCMs at 2.5 arc-minute resolution. Predictor variables used were mean annual temperature, mean diurnal range, temperature seasonality, minimum temperature of coldest month, annual precipitation and precipitation seasonality. Binary maps for each model were created using the maximum sensitivity plus specificity logistic threshold. The resulting binary grids were used to evaluate species range overlap with agricultural frontiers in current climate and in all future climate scenarios.

## Results

### Extent of climate-driven agricultural frontiers

**Fig 1:**
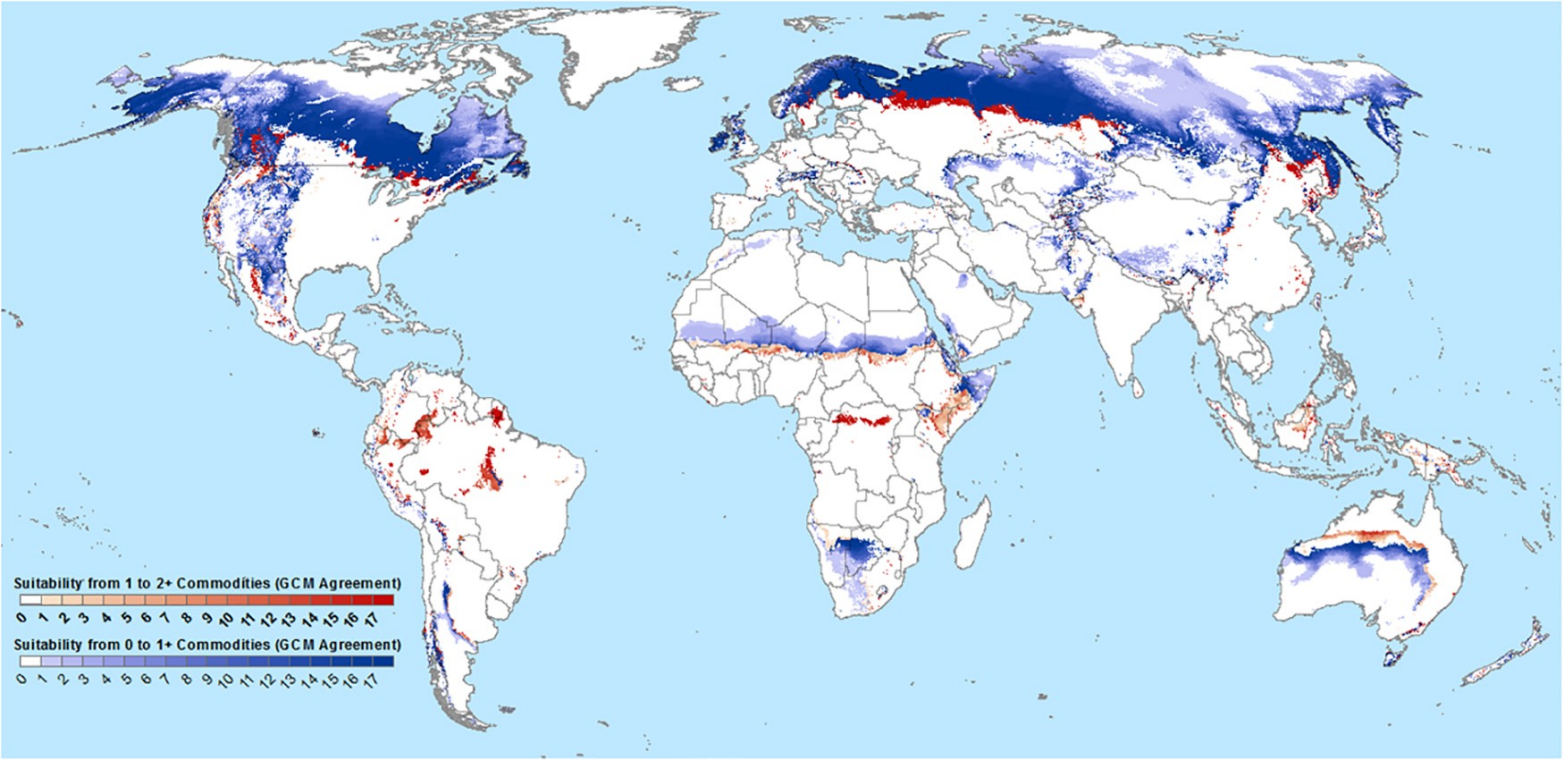
Global climate-driven agricultural frontiers for RCP8.5 2060–2080. Areas that transition from no current suitability for major commodity crops to suitability for one or more crops are depicted in blue, while currently uncultivated areas that transition to suitability for multiple major commodity crops are shown in red. Intensity of color indicates the level of agreement between simulations driven by different GCMs for the RCP 8.5 radiative concentration pathway. Terrestrial areas in white are either currently suitable for at least one modeled crop or, not suitable for any modeled crops in the projected climatic conditions. Suitability under current and projected climates is defined as universal agreement of suitability methods (EcoCrop, Maxent, Frequency of Extreme Temperatures).

Climate-driven agricultural frontiers as defined here cover between 10.3–24.1 million km^2^ of the planet’s surface, with an ensemble median value of 15.1 million km^2^ under RCP 8.5 by 2060–2080 (Fig 1, S2 Fig). Crops that comprise the frontiers are shown in supplementary S3 and S4 Figs and are primarily more cold tolerant temperate crops such as potatoes, wheat, maize, soy. To put the magnitude of these agricultural frontiers in perspective, the ensemble median area of agricultural frontiers under this late century, RCP 8.5 scenario is equivalent to 59% of current global cultivated and managed vegetation land area, while the ensemble maximum area is equivalent to 93% of current cultivation. Under a RCP 4.5 scenario, with more muted radiative forcing, agricultural frontiers are found to cover 8.1–20.0 million km2 of the earth’s surface (equivalent to 31–77% of currently cultivated area (see S2 Fig)). Soil quality, terrain and infrastructure, however, will be major determinants of which of these frontiers will actually be cultivated and as such, the results presented here represent an upper bound estimate of where cropland expansion may be expected.

### Geographic distribution of climate-driven agricultural frontiers

Frontiers are projected to be most extensive in the boreal regions of the Northern Hemisphere and in mountainous areas worldwide, since areas suitable for commodity production generally expand upslope and towards the poles in response to rising temperatures. Potatoes, wheat and maize make the largest contributions to frontier land surface (S4 Fig). Canada (4.2 million km^2^) and Russia (4.3 million km^2^) harbor the greatest area of agricultural frontier (RCP 8.5, ensemble median). Among montane regions, the Mountains of Central Asia and the Rocky Mountains of USA and Canada have the greatest frontier area (0.1 and 0.9 million km^2^, respectively). Frontiers on the fringes of Australian and African deserts are the result of projected increases in precipitation, for which there is relatively low GCM agreement, including divergent trends in sign of precipitation change among GCMs. This makes conclusions about potential for agricultural expansion in these areas highly uncertain. In contrast, there will be a small loss of existing crop area. We estimated that about 0.2% of existing crop area will become unsuitable for all modelled crops without irrigation or other intensive inputs for RCP 8.5 2060–2080 scenario.

### Environmental impacts of climate-driven agricultural frontiers

Environmental impacts from climate-related agricultural land use change include impacts on climate services (e.g., reduction in carbon storage), the effects of agricultural pollution on downstream areas, and degradation of natural habitats with attendant loss of biodiversity [47–52]. The most significant impact is likely reduction in climate services provided by carbon storage in frontiers soils, particularly in the extensive high latitude frontiers.

### Climate services impact

**Fig 2:**
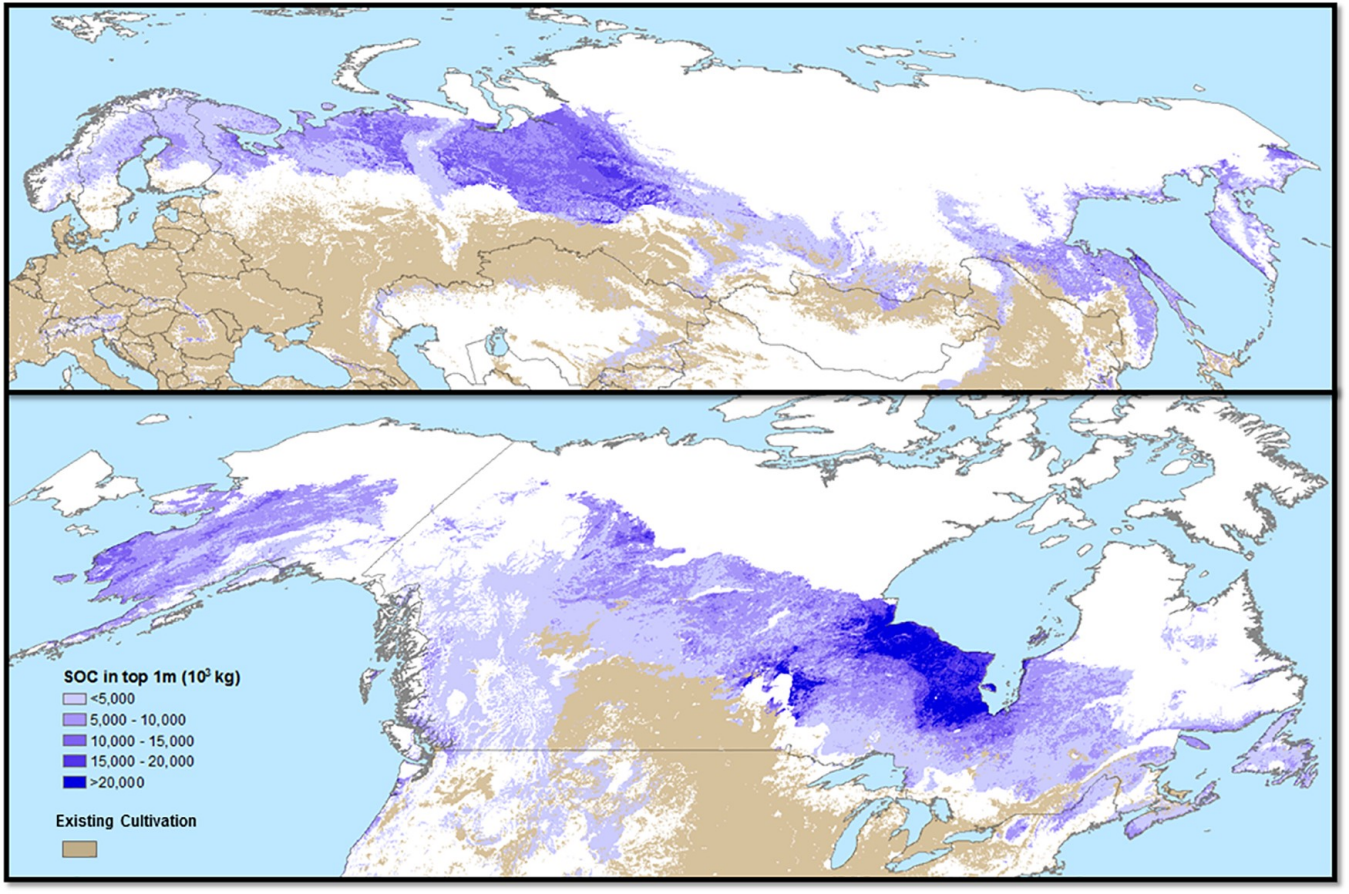
Soil organic carbon content in top 1 meter of soil (10^3^ kg ha-1) in areas of climate-driven agricultural commodity frontiers using RCP8.5 2060–2080 climate projections (blue color ramp). Areas with >50% GCM agreement commodity frontiers are shown. Existing agricultural land cover >10% of each pixel is represented in light brown.

The total amount of carbon that resides in the top 1 m of soil under agricultural frontiers has a median value of 632 GtC (gigatons of carbon) (RCP 8.5, ensemble) and 539 GtC (RCP 4.5, ensemble), with a minimum RCP 8.5 ensemble value of 400 and a maximum value of 991 GtC (Table 2). This is equivalent to 47–116% of all carbon currently in the Earth’s atmosphere (Fig 2). Release of carbon from high latitude soils due to warming is already of major concern but may be small relative to the amounts of carbon that might be released if these areas come under cultivation [53].

**Table 2:** Accounting of soil organic carbon in top 1m in areas of agricultural frontiers and resulting potential carbon emissions under RCP 8.5 and RCP 4.5. Rows with grey shading apply GAEZ general soil suitability constraints and soil requirements for each crop to the climatically suitable frontier areas.

Landcover classification	Soil organic carbon stock (GT) 1m	Low-end IPCC method estimate C release (GT) *[forest 25%; grass/shrubland 10%; wetlands 20%]*	High-end IPCC method estimate C release (GT) *[forest 25%; grass/shrubland 10%; wetlands 110%]*	1m estimate C release (GT) [*all land classes 25%]*	1m estimate C release (GT) [*all land classes 40%]*
Forests (RCP 8.5)	267.5 *[192*.*6–355*.*4]*	66.9 *[48*.*1–88*.*9]*	66.9 *[48*.*1–88*.*9]*	66.9 *[48*.*1–88*.*9]*	107.4 *[77*.*0–142*.*2]*
	243.3 [174.6–328.6]	60.8 *[43*.*7–82*.*2]*	60.8 *[43*.*7–82*.*2]*	60.8 *[43*.*7–82*.*2]*	97.3 *[69*.*9–131*.*5]*
Shrubland/Grassland (RCP 8.5)	286.6 *[133*.*6–564*.*0]*	28.7 *[13*.*4–56*.*4]*	28.7 *[13*.*4–56*.*4]*	71.6 *[33*.*4–141*.*0]*	114.6 *[53*.*5–225*.*6]*
	265.9 *[118*.*7–532*.*5]*	26.6 *[11*.*9–53*.*3]*	26.6 *[11*.*9–53*.*3]*	66.5 *[29*.*7–133*.*1]*	106.4 *[47*.*5–213*.*0]*
Permanent wetlands (RCP 8.5)	73.6 *[56*.*6–74*.*3]*	14.7 *[11*.*3–14*.*9]*	81.0 *[62*.*3–81*.*7]*	18.4 *[14*.*2–18*.*6]*	29.4 *[22*.*7–29*.*7]*
	54.2 *[54*.*8–38*.*5*]	10.8 *[7*.*7–11*.*0]*	60.3 *[42*.*3–59*.*6]*	13.5 *[9*.*6–13*.*7]*	21.7 *[15*.*4–21*.*9]*
Total soil organic carbon (RCP 8.5)	632.4 *[400*.*0–991*.*5]*	110.2 *[72*.*8–160*.*1]*	176.5 *[123*.*8–226*.*9]*	158.1 *[100*.*0–247*.*9]*	251.1 *[153*.*1–397*.*5]*
	569.2 *[347*.*6–914*.*0]*	98.2 *[63*.*2–146*.*4]*	147.0 *[97*.*8–195*.*7]*	142.3 *[86*.*9–228*.*5]*	225.3 *[132*.*7–366*.*4]*
Forests (RCP 4.5)	271.9 *[197*.*4–353*.*8]*	68.0 *[49*.*3–88*.*4]*	68.0 *[49*.*3–88*.*4]*	68.0 *[49*.*3–88*.*4]*	109.8 *[78*.*9–141*.*5]*
	248.3 [178.9–326.7]	62.1 *[44*.*7–81*.*7]*	62.1 *[44*.*7–81*.*7]*	62.1 *[44*.*7–81*.*7]*	99.3 *[71*.*5–130*.*7]*
Shrubland/Grassland (RCP 4.5)	196.5 *[77*.*2–434*.*9]*	19.7 *[7*.*7–43*.*5]*	19.7 *[7*.*7–43*.*5]*	49.1 *[19*.*3–108*.*7]*	78.6 *[30*.*9–174*.*0]*
	178.1 *[67*.*6–401*.*3]*	17.8 *[6*.*8–40*.*1]*	17.8 *[6*.*8–40*.*1]*	44.5 *[16*.*9–100*.*3]*	71.2 *[27*.*1–160*.*5]*
Permanent wetlands (RCP 4.5)	71.5 *[39*.*6–74*.*3]*	14.3 *[7*.*9–14*.*9]*	78.6 *[43*.*6–81*.*7]*	17.9 *[9*.*9–18*.*6]*	28.6 *15*.*9–29*.*7]*
	52.4 *[26*.*2–54*.*8]*	10.5 *[5*.*2–11*.*0]*	57.7 *[28*.*9–60*.*3]*	13.1 *[6*.*6–13*.*7]*	21.0 *[10*.*5–21*.*9]*
Total soil organic carbon (RCP 4.5)	539.9 *[314*.*2–862*.*9]*	101.9 *[65*.*0–146*.*8]*	166.2 *[100*.*7–213*.*6]*	135.0 *[78*.*5–215*.*7]*	215.9 *[125*.*7–345*.*2]*
	470.8 *[272*.*8–771*.*3]*	90.4 *[56*.*7–132*.*8]*	137.5 *[80*.*3–182*.*1]*	119.7 *[68*.*2–195*.*7]*	191.5 *[109*.*1–313*.*1]*

The release of carbon following tilling from previously untilled soils is believed to occur rapidly and estimates suggest that 25–40% of total soil carbon is released within five years of plowing [54]. Therefore, an upper bound estimate of the total amount of carbon that might be released from the cultivation of climate-driven frontiers would be on the order of 177 GtC, which is equivalent to 119 years of current CO_2_ emissions of the United States [55]. The actual area affected would be smaller than the frontier due to economic and physical factors, but emissions might be greater because many of the potentially affected soils are peat, which may degrade when disturbed, releasing more and deeper carbon. In either event, the magnitude of the potential release indicates that policies directed at constraining development of these areas are vitally important. From a global perspective, 177 GtC is more than two-thirds of the 263 GtC within which total future emissions must be constrained to limit global mean temperature increase to the internationally agreed Paris agreement target of 2°C global mean temperature increase above pre-industrial levels [56].

One way to address the challenge posed by cultivation of frontiers is through promoting agricultural management practices that conserve soil-bound carbon. In particular, policies that incentivize leaving peat soils intact and promoting conservation tillage could significantly reduce the quantity of carbon released and slow the speed at which it is released [57–58]. Thus, while specific estimates as to the speed or extent to which these carbon sources might affect the atmosphere is beyond the scope of this study, it is highly likely that developing such regions for agriculture will have significant impacts on greenhouse gas emissions that need to be balanced against the benefits of increased food supply and constrained by sound environmental policies.

### Biodiversity impacts

The biodiversity impacts of the climate-driven frontiers occur where the frontiers intersect with important ecosystems and habitats (Table 3). Among global priorities for biodiversity conservation, 56% of global biodiversity hotspots, 22% of Endemic Bird Areas (EBAs) and 13% of Key Biodiversity Areas (KBAs) intersect with climate-driven agricultural frontiers (ensemble median RCP8.5 2060–2080; see Table 3). Biodiversity hotspots that have the largest intersection with frontiers are the Tropical Andes, the Mountains of Central Asia, the Horn of Africa and the Chilean Winter Rainfall and Valdivian Forests.

**Table 3:** Environmental impacts of agricultural frontiers under both RCP8.5 and RCP4.5 2060–2080 climate projections. Areas of significant biodiversity resources assessed are biodiversity hotspots; endemic bird areas (EBA); key biodiversity areas (KBA). Numbers presented for biodiversity resources are the median [range] number of areas that intersection with frontiers across all GCMs. Potential impacts on restricted range bird species are presented as the median [range] number of species with modeled range intersection with frontiers in current and 2060–80 climate projection. Modeled future ranges are assessed under an assumption of no-dispersal and a 10 km/decade dispersal rate.

Global Conservation Priority/Ecosystem Service	Potential Impact of Climate-Driven Agricultural Frontiers	
**Important Biodiversity Areas**	**RCP8.5**	**RCP4.5**
Biodiversity Hotspots (n = 34)	19 *[18–22]*	19 [17–21]
Endemic Bird Areas (n = 218)	48 *[43–52]*	49 [43–54]
Key Biodiversity Areas (n = 11,824)	1590 *[1361–1810]*	1601 [1416–1788]
**Restricted Range Birds**		
Current Range	409 *[375–436]*	409 [380–442]
2060–80—No Dispersal	385 *[344–410]*	228 [185–295]
2060–80—Full Dispersal	491 *[452–525]*	362 [294–432]

Species’ ranges may move in response to climate change, causing changes in patterns of biodiversity, at the same time as frontiers are opening. To test the effect of frontiers on future, as well as present, patterns of biodiversity, the ranges of all global restricted range birds, a set of high conservation priority species found in hotspots, KBAs and EBAs, were modelled [59]. These results show that the number of restricted range birds impacted by frontiers increases in the future from 409 species to 491 species under RCP8.5 representing 20% of the 2,451 global restricted range bird species and 409 to 362 under RCP4.5 (Table 3). Thus, range shifts due to climate change accentuate the intersection of frontiers with suitable climate for rare species, as both crop suitability and suitable climate for species move upslope. However, this effect depends on species’ ability to occupy newly suitable areas. Species potentially impacted by frontiers are most numerous in Central America and the Northern Andes, with secondary concentrations in the Himalayas and highlands of New Guinea.

### Water quality impacts

The potential impact of climate-driven agricultural frontiers on downstream water quality has may affect large numbers of people and their water infrastructure. The agricultural water quality (AWQ) footprint of frontiers encompasses the homes of between 0.4–1.0 billion people (RCP 4.5, ensemble minimum and maximum) and 1.2–1.8 billion people (RCP 8.5, ensemble minimum and maximum), of whom 900 million-1.6 billion (RCP 8.5, ensemble minimum and maximum) live in areas in which more than half of the water supply is projected to be impacted (Table 4). Water quality changes in these downstream areas from fertilizer and biocide runoff may affect human health, ecosystem health, production of fisheries and the cost of water treatment.

**Table 4:** Agricultural water quality (AWQ) impact of climate-driven agricultural frontiers under RCP 8.5 and RCP 4.5. Elevated AWQ is >50% of water supply with AWQ impacts.

	AWQ footprint (million km^2^)	Elevated AWQ^1^ footprint (million km^2^)	Current population affected by AWQ (billion)	Current population affected by elevated AWQ^1^ (billion)	Potential population affected by AWQ (billion)	Potential population affected by elevated AWQ (billion)	Footprint per land area of cropland	Global dam estate affected by AWQ (%)	Global dam estate affected by elevated AWQ^1^ (%)
Frontiers RCP 8.5 2060–2080 Median	11.7	5.2	0.7	0.1	1.4	1.1	0.79	7.3	2.9
Frontiers RCP 8.5 2060–2080 Max	15.6	7.8	0.8	0.2	1.8	1.6	0.87	8	3.8
Frontiers RCP 8.5 2060–2080 Min	9.1	3.9	0.7	0.1	1.2	0.9	0.67	6.4	2
Frontiers RCP 4.5 2060–2080 Median	6.6	2.2	0.5	0.07	0.7	0.662	0.82	6.3	2.4
Frontiers RCP 4.5 2060–2080 Max	8.9	3.6	0.52	0.1	1.017	1.017	0.95	6.9	3.1
Frontiers RCP 4.5 2060–2080 Min	4.7	1.2	0.4	0.05	0.407	0.407	0.65	5.5	1.7
Global (reference period 1960–90)	53.2	37.6	7.5	4.1	9.4	7.5	0.71	63.3	50.2

Table 4 shows that agricultural frontiers increase the amount of land potentially affected by changes in AWQ 9% to 16% (median 12%) compared to current impact (RCP 8.5, ensemble minimum and maximum). Given that some of this new farmland (in drylands) will not generate significant runoff, under RCP 8.5 the land area with AWQ varies from a maximum additional 7–12% (median 9%) with the maximum additional global population affected varying from 9–10% (median 9%). Elevated levels of AWQ (>50%), affecting 3–6% of additional land surface (median 4%), impacting an additional 2–3% (median 2%) of the current global population. The additional AWQ per unit land area of new cropland varies between ensemble members and reflects the distribution of cropland in runoff generating areas vs not, as well as the downstream differential mixing of runoff from agricultural and non-agricultural land under different spatial frontier outcomes.

Hydrologic infrastructure, including the global estate of reservoirs created by dams that are essential for urban water supply, irrigation and hydropower are also potentially affected by the AWQ footprint of agricultural frontiers. 6.4–8% (median 7.3%, RCP8.5) or 5.5–6.9% (median 6.3% RCP4.5) of global reservoirs would experience increased AWQ impacts as a result of agricultural frontiers and 2.0–3.8% (median 2.9%, RCP 8.5) or 1.7–3.1% (median 2.4% RCP4.5) of reservoirs would be exposed to elevated impacts (AWQ >50%). These are in addition to the 63.3% of reservoirs already with AWQ>0 under the current distribution of crop suitability (50.2% at >50% AWQ) (Table 4).

### Uncertainty

To account for the uncertainty of future climate projections, all impacts of climate driven agricultural frontiers were assessed on an individual GCM/RCP/time period basis and results are presented as ensembles across all climate projections. The choice of binary threshold is a possible source of uncertainty, but in this analysis that uncertainty is constrained by choosing a threshold that is conservative from the perspective of frontiers. For instance, in EcoCrop a threshold of 20 (“very marginal to marginal”) includes areas that are possible but not optimal for cultivation [22–23]. The total area of frontiers is largely insensitive to adjustments to the choice of threshold across all methods used, because under a more permissive threshold the currently suitable area will expand, but there will be an accompanying expansion of frontiers poleward—and vice versa for a less permissive threshold. Uncertainty is more difficult to constrain in precipitation-driven frontiers where there is high disagreement on sign of change in GCMs. This makes Sahelian and Australian precipitation-driven frontiers much more uncertain than other frontiers, as noted above. The greatest uncertainty is in actual cultivation of frontiers, as discussed below. Comparison of the modeled crop distributions for both current and future climates including the possible reduction of frontier areas due to soil constraints as defined by the union of GAEZ soil resource classifications are shown in S5–S8 Figs.

## Discussion

This paper has three specific implications as well as raising some broader scale issues that need to be reflected on. In terms of the specific implications, we note that while climate change is creating new opportunities for agriculture, especially in northern latitudes, results presented here show huge potential environmental trade-offs. The first potential impact relates to the release of soil organic carbon into the atmosphere. As shown in the results section of this paper, if agriculture is allowed to extend into all of the frontiers identified here, then there would be little chance of reaching the Paris climate accord’s goals of keeping climate change to within 1.5°C above preindustrial levels [60]. A second implication relates to biodiversity. The results of this paper suggest that if all of the frontiers are converted into agricultural uses, the world will lose important biodiversity hotspots in both mountainous and northern regions. This will accentuate the negative impact of what some scholars describe as “the Anthropocene” [61]. This paper shows that a third implication of developing all of these agricultural frontiers would be significant problems of water degradation that could affect the health and well-being of millions of people.

With that said, it is important to acknowledge that the analysis presented here does not assess economic incentives and constraints on frontier development, nor does it simulate the probability that individual areas would be cultivated. Consequently, the carbon, water and biodiversity impacts described here are all “upper bound” estimates and in some ways represent worst case scenarios. However, the magnitude of impacts identified, and the potential for very significant feedbacks in terms of environmental problems (such as cultivating the Northern frontier leading to increased carbon emissions leading to more rapid climate change), should trigger concern. In this way, the results presented here should be seen alongside literature on biogeophysical “tipping cascades” that could push the Earth System across a planetary threshold to a ‘Hothouse Earth’ pathway [61]. We have identified climate-driven agricultural frontiers as a coupled natural-human tipping cascade that might have similar self-reinforcing tendencies. In lieu of more detailed analyses, there are several reasons to believe that frontier development, particularly in the Northern Hemisphere high latitude frontiers, is of both global and regional policy significance.

Standing aside from these ecological issues, these results also have implications for society and mean that this analysis makes a contribution to the broad area of sustainability science and the emerging literature on planetary boundaries and planetary health [62–63]. In particular, there are serious social issues that would need to be considered that fall outside of the scope of this paper. A great many First Nations communities call these areas home and have ancestral claims many of which are unseeded. The development of any agricultural “frontier” would, therefore, need to be done fully cognizant of the fact that Indigenous Communities must be at the forefront of any development plans and must be the primary beneficiaries. Any such developments, therefore, must take into account a number of the following.

First, humanity has a history of cultivating land that was once deemed unlikely to ever justify cultivation–and this has created massive sustainability problems. For example, a combination of policies and technology created conditions that led to the Dust Bowl of the 1930s in North America [64]; or that 60 years ago resulted in the beginning of the degradation of the Aral Sea and the pollution of its waters, with knock-on effects on the very same ability of lands to sustain agricultural productivity [65]. Similarly, 50 years ago, no one seriously thought that people would ever turn large areas of the Brazilian Cerrado and Amazon into soy fields or large areas of Southeast Asian peat into oil palm plantation. In particular, the fragile tropical soils were deemed unsuitable and the areas were considered too remote. Yet land use conversion happened in a very short period of time driven by rising demand and low land prices. Arguing by analogy, it seems plausible that we should be prepared for a similar economic logic to be applied to the global North. For instance, policies such as China’s “Belt and Road” initiative, are likely to provide significant subsidies to frontier development. Adding environmental policies within such programs could help reduce the impacts associated with frontier development, for instance by promoting non-agricultural industries and supporting low carbon forms of land use.

Second, we have evidence that populations are already looking north for food producing opportunities. For example, the government of the Northwest Territories in Canada recently created a new agricultural strategy that promotes development of northern lands [66]. Similarly, Russia has policies promoting homesteading in Siberia that will attract more settlers as warming continues, while China and Korea have both leased land in Siberia for agriculture even under current climatic limitations [67–68].

Third, there is technology change. New genetically modified crops, including quick maturing soybeans, and new management practices, such as precision agriculture, are giving farmers the ability to plant in environments that once would have been considered extreme. As a result, the frontier for soybeans in North America has been moving west and north for years. It is important to bear in mind that, therefore, today it seems that the Northern agricultural frontier is at a moment in history similar to just before Brazil started investing in soy production [69–70].

Finally, population growth and the expansion of biofuels may have outsized impacts on land use. Biofuel use is strongly influenced by national and regional (e.g., EU) policies, which can change. Policy change could drastically increase land requirements for biofuel production, as it has done in Brazil. It is important to recognize the land use consequences, such as in agricultural frontiers, of such policies. Global population growth estimates diverge strongly after 2050. After 2050, global population estimates range from decline to just over 7 billion people (low-variant) to more than doubling to over 16 billion people (high-variant) [71]. The upper-range population endpoints, while less likely, would result in very different demand drivers for new agricultural production in our end-century scenarios. The environmental consequences of frontier development are one of many reasons we should be concerned about which of these trajectories the planet will follow.

The distribution of both the benefits and the impacts of frontier development will further complicate achievement of the targets set by the sustainable development goals [72–73]. In particular, although developing the Northern frontiers might help reduce poverty and hunger both through the economic activities as well as the food produced in these areas, developing such frontiers might have a detrimental effect on the Sustainable Development Goals of the United Nations (13 climate action 14 life in water and 15 life on land). Further development imbalances may be created by distributional effects caused by the development of the frontiers. Namely, it must be noted that two countries–Canada and Russia–contain 56% of the global frontier area. Frontier cultivation may have significant economic, food security and trade benefits for these countries, providing significant incentives favoring development. However, the likely environmental cost, especially related to climate change, will be felt internationally, with disproportionate impact on poor nations [74*–75*].

Overall, therefore, climate-driven agricultural frontiers pose a major challenge for international environmental policy. In particular, changing crop suitability in the frontiers is likely to be a gradual, but sustained, source of new greenhouse gas emissions that may make it difficult for some countries to make progressive reductions toward Paris Agreement targets.

## Conclusion

In summary, our research shows that climate change presents serious opportunities for food production in areas that, until now, have been relatively undeveloped. This suggests opportunities for economic development that, if done properly, may reduce poverty and food insecurity in some economically marginal parts of the world, such as northern Canada, where a lack of economic opportunities has created epidemic levels of food insecurity. With that said, it is important to recognize that food insecurity in remote communities is rarely a function of food production alone and is more often associated with a complex legacy of colonialism, education, and socio-cultural disconnects.

There are serious negative environmental impacts associated with the unfettered development of climate-driven agricultural frontiers. Recognizing that climate-driven agricultural frontiers are a potential source of new greenhouse gas emissions and other environmental impacts including a loss of biodiversity and a loss of water quality for hundreds of millions of people highlights the need for national and international policy to guide sustainable development in the frontiers. The development of such policies should engage with Indigenous Communities and other local stakeholders to establish participatory processes that would ensure that economic development plans are led locally and that local communities are the primary beneficiaries of any land use change. Together, therefore, using participatory methodologies, local governance and frameworks such as the Sustainable Development Goals, it should be possible to help countries realize the potential benefits associated with a changing environment without causing major further environmental problems.

## Supporting information

S1 Table List of GCMs used to drive future climate projections. (DOCX)

S2 Table Definition of bioclimatic variables used in maxent models. (DOCX)

S3 Table List of crops modeled and ecocrop parameters used. KTmp = Killing Temperature (°C); Tn = Absolute Minimum Temp (°C); TnOp = Minimum Optimal Temp (°C); Tx = Absolute Maximum Temp (°C); TxOp = Maximum Optimal Temp (°C); Pn = Absolute Minimum Precip (mm); PnOp = Minimum Optimal Precip (mm); Px = Absolute Maximum Precip (mm); PxOp = Maximum Optimal Precip (mm); Gseas = Growing Season Duration (days).
(DOCX)

S4 Table Gridded datasets used to derive occurrence points for each crop. (DOCX)

S5 Table Bioclimatic variables used to create the maxent model for each crop. (DOCX)

S6 Table Summary of model performance (AUC), logistic threshold used to create binary distributions (Threshold) and the four most important variables for each crop (Var 1–4). (DOCX)

S7 Table Critical maximum and minimum temperatures for each crop. Critical temperature thresholds used are in bold. Critical temperature values were obtained from references [76–90].
(DOCX)

S8 Table Dice-Sorenson spatial congruence for ecocrop, maxent, and combined model with extreme temperature masks. Dice-Sorenson measures the spatial overlap with the formula 2a/2a+b+c where a = cells in common and b + c = cells in disagreement.
(DOCX)

S1 Fig Flowchart showing the input data, modeling methods, frontier determination and impact analysis. Sources for data are indicated by reference number.
(TIF)

S2 Fig Global map of climate-driven agricultural frontiers. Blue color ramp shows transition from zero currently suitable commodity crops to one or more suitable commodities. Red color ramp shows transition from one suitable commodity to two or more suitable commodities. Intensity of color denotes level of GCM agreement for the RCP8.5 2040–2060 climate scenario. Areas in grey are either currently suitable for two or more commodities or not suitable for any commodities in the projected climate.
(TIF)

S3 Fig GCM agreement (green = low; blue = high) of modeled frontiers for individual commodity crops for RCP 8.5 2060–2080. (TIF)

S4 Fig Distribution across 17 GCMs of total area (million km^2^) in commodity frontiers for each modeled crop under RCP8.5 2060–2080 climate projections. Boxes are interquartile range; whiskers extend to 1.5 times interquartile range; outliers indicated by points.
(TIF)

S5 Fig Comparison of modeled agricultural frontiers with areas of very severe soil constraints (green). Soil constraints were defined as the union of GAEZ soil resource classifications and crop specific limitations of pH and soil depth. Areas in green were used to reduce total frontier impact on soil carbon in Table 2.
(TIF)

S6 Fig Comparison of agricultural frontiers of this paper (blue and red) vs. MAGPIE RCP SSP-5 [91] cropland in 2070 (green). (TIF)

S7 Fig Comparison of modeled wheat suitability in available GAEZ gridded datasets (blue—Top panel) and this paper (red—Bottom panel). An exact match for GCM, scenario and time step was not available, but shows general spatial congruence of the modeling methods for late-century, high emissions scenarios. Existing agriculture is represented in grey for both panels.
(TIF)

S8 Fig Comparison of existing cultivated land (green) with modeled current suitability (purple) and soil constraints (black overlay). Areas of visible purple are where the universal agreement among three models indicates that it is currently suitable for at least one modeled commodity crop but is not currently cultivated. The pole ward edges of the extent of modeled vs. realized agriculture are in good agreement, with modeled agriculture extending further than existing continuous agriculture. Quantification of frontiers and their potential impacts presented here are therefore conservative.
(TIF)
