# Correction: The environmental consequences of climate-driven agricultural frontiers

**DOI:** 10.1371/journal.pone.0236028

**Published:** 2020-07-08

**Authors:** The PLOS ONE Staff

The third author’s name is spelled incorrectly. The correct name is: Krishna Bahadur KC. The correct citation is: Hannah L, Roehrdanz PR, KC KB, Fraser EDG, Donatti CI, Saenz L, et al. (2020) The environmental consequences of climate-driven agricultural frontiers. PLoS ONE 15(2): e0228305. https://doi.org/10.1371/journal.pone.0228305. The publisher apologizes for the error.
