# Supplementary figures and images for: The environmental consequences of climate-driven agricultural frontiers
Source: PLoS One. 2020 Feb 12;15(2):e0228305. doi: 10.1371/journal.pone.0228305 (PMC7015311; doi:10.1371/journal.pone.0228305)

**Table S1. List of GCMs used to drive future climate projections**


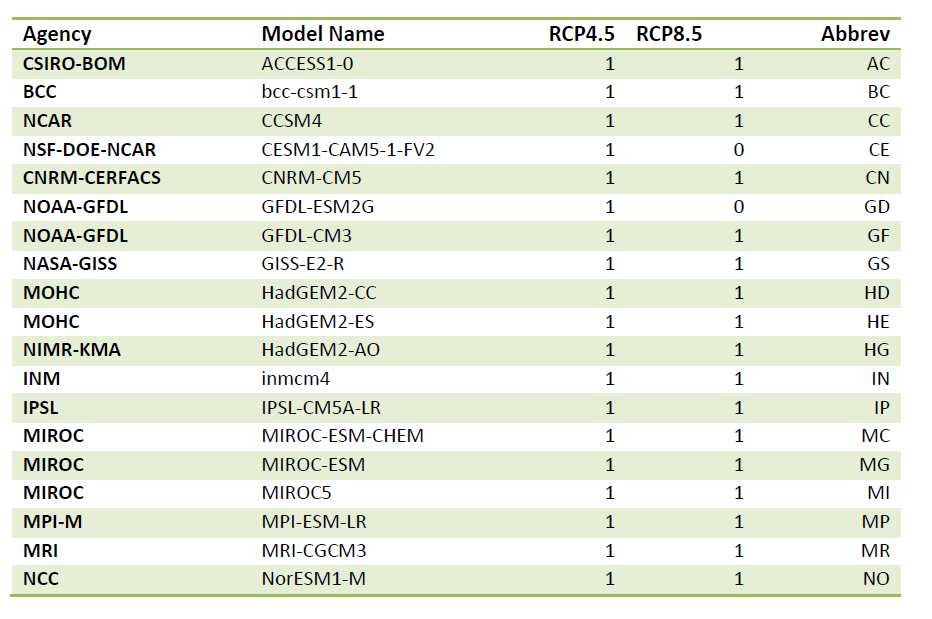

Supplement: S1 Table — (DOCX) [file pone.0228305.s001.docx]

**Table S2**. **Definition of Bioclimatic variables used in Maxent models**


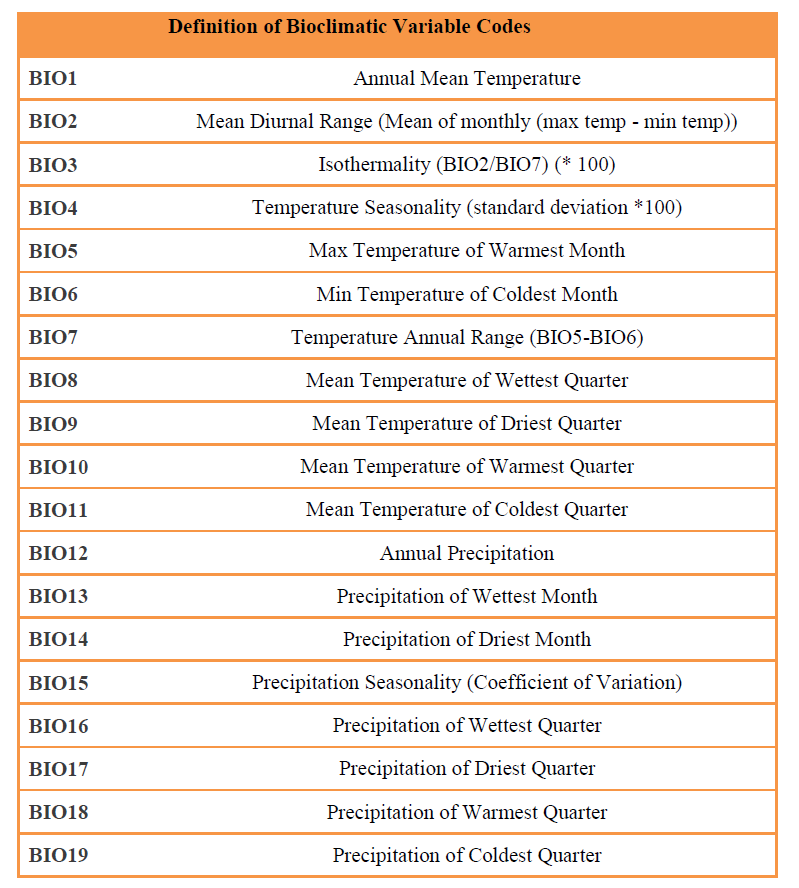

Supplement: S2 Table — (DOCX) [file pone.0228305.s002.docx]

**Table S4. Gridded datasets used to derive occurrence points for each crop**


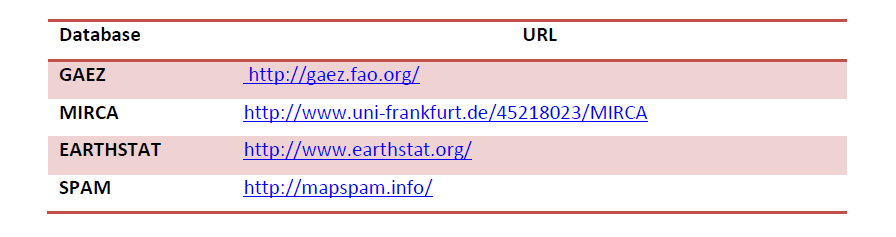

Supplement: S4 Table — (DOCX) [file pone.0228305.s004.docx]

**Table S5. Bioclimatic Variables used to create the Maxent model for each crop.**


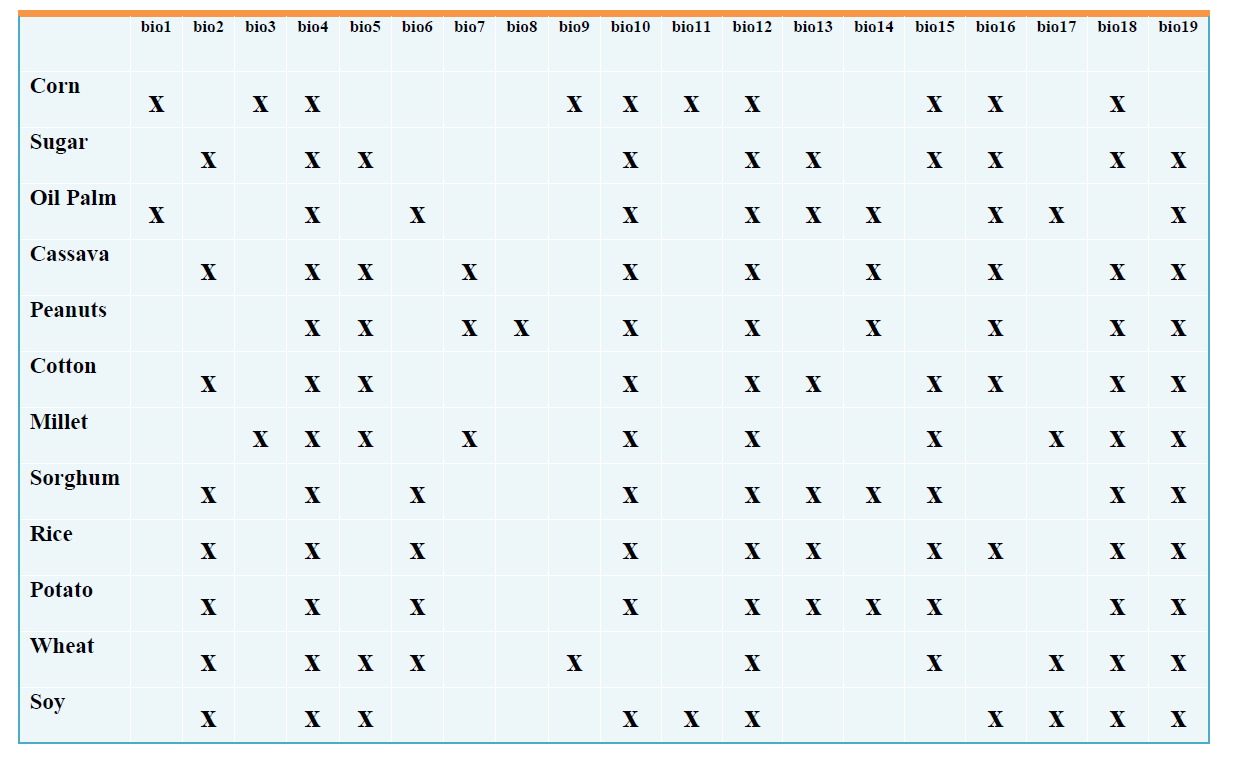

Supplement: S5 Table — (DOCX) [file pone.0228305.s005.docx]

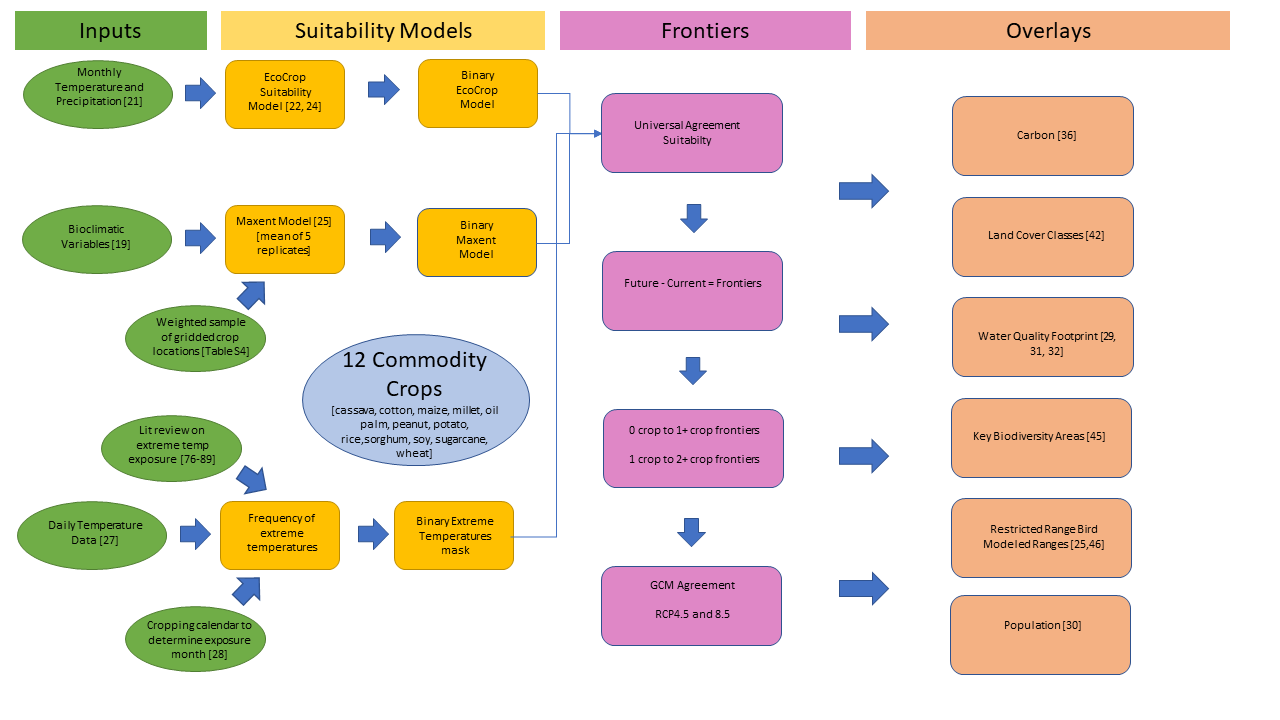

Supplement: S1 Fig — Sources for data are indicated by reference number. (TIF) [file pone.0228305.s009.tif]

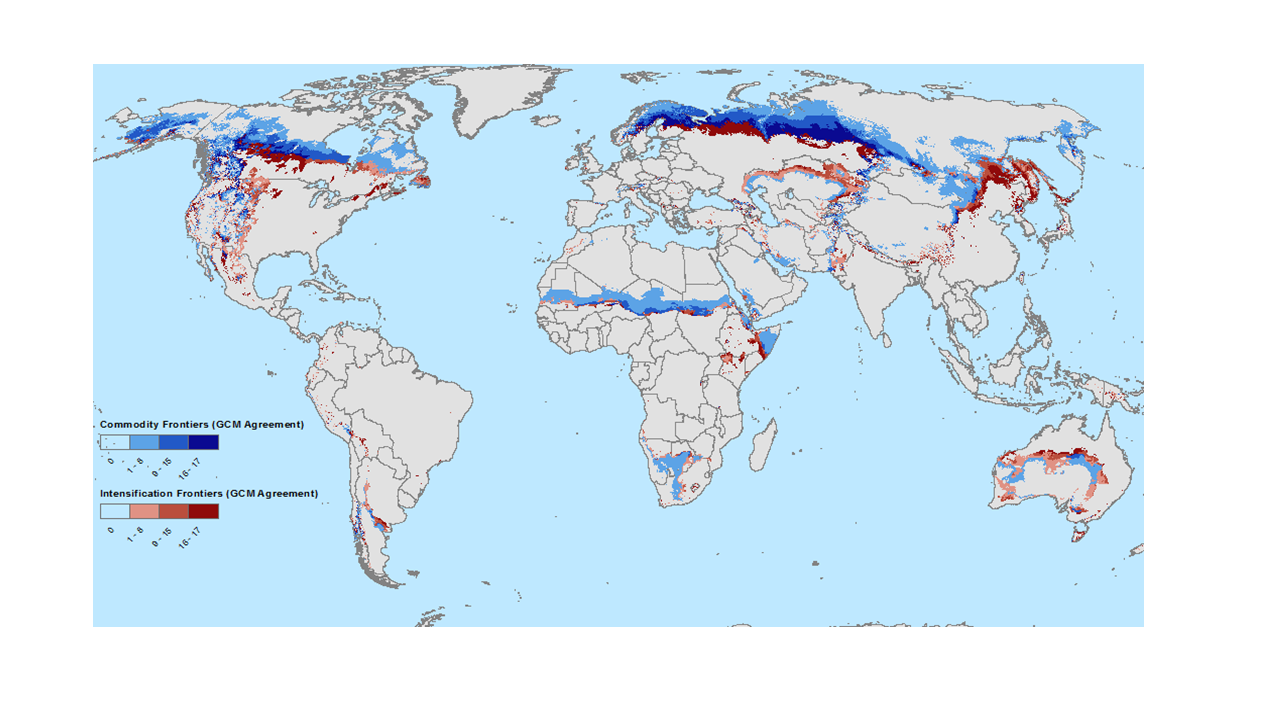

Supplement: S2 Fig — Blue color ramp shows transition from zero currently suitable commodity crops to one or more suitable commodities. Red color ramp shows transition from one suitable commodity to two or more suitable commodities. Intensity of color denotes level of GCM agreement for the RCP8.5 2040–2060 climate scenario. Areas in grey are either currently suitable for two or more commodities or not suitable for any commodities in the projected climate. (TIF) [file pone.0228305.s010.tif]

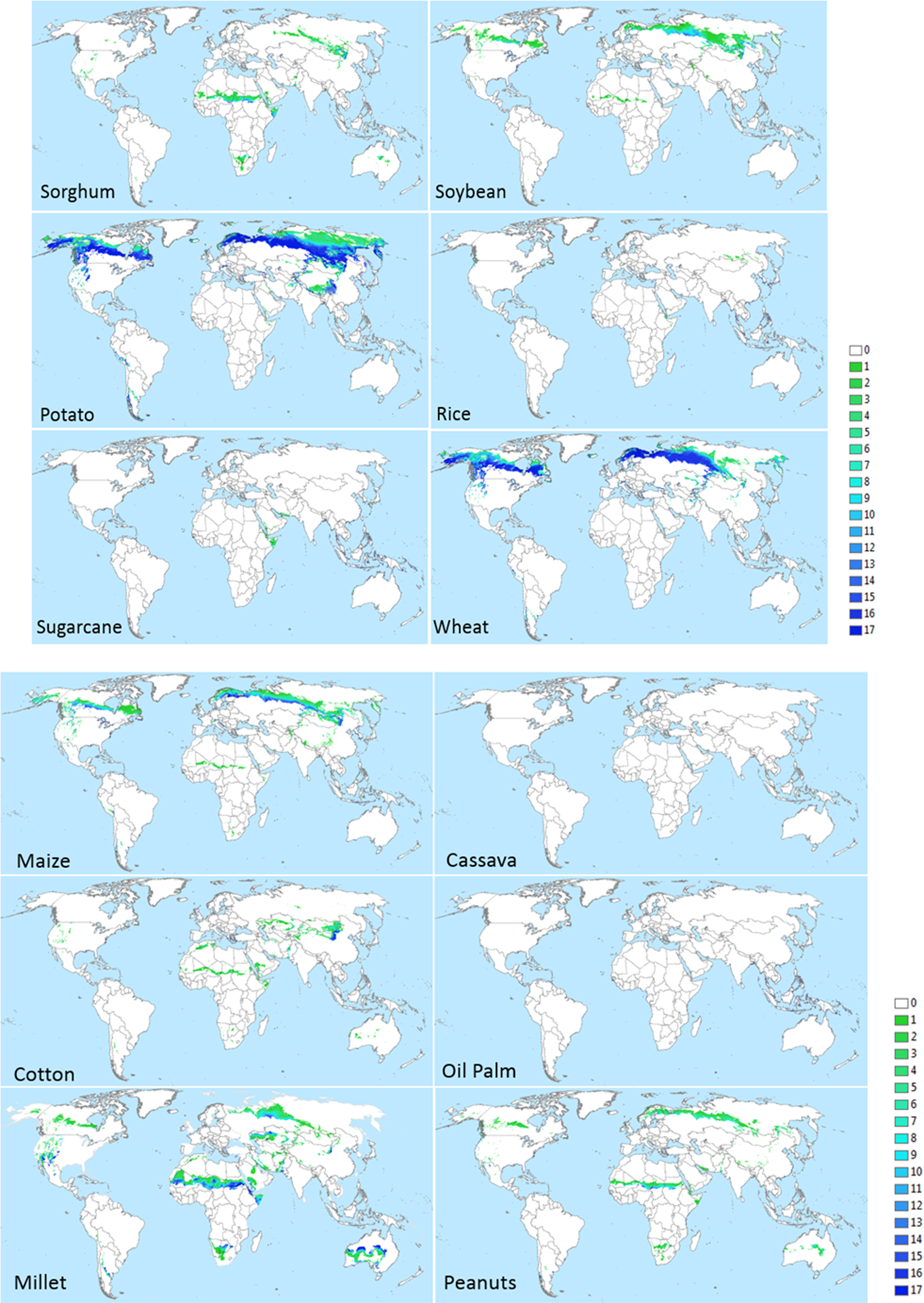

Supplement: S3 Fig — (TIF) [file pone.0228305.s011.tif]

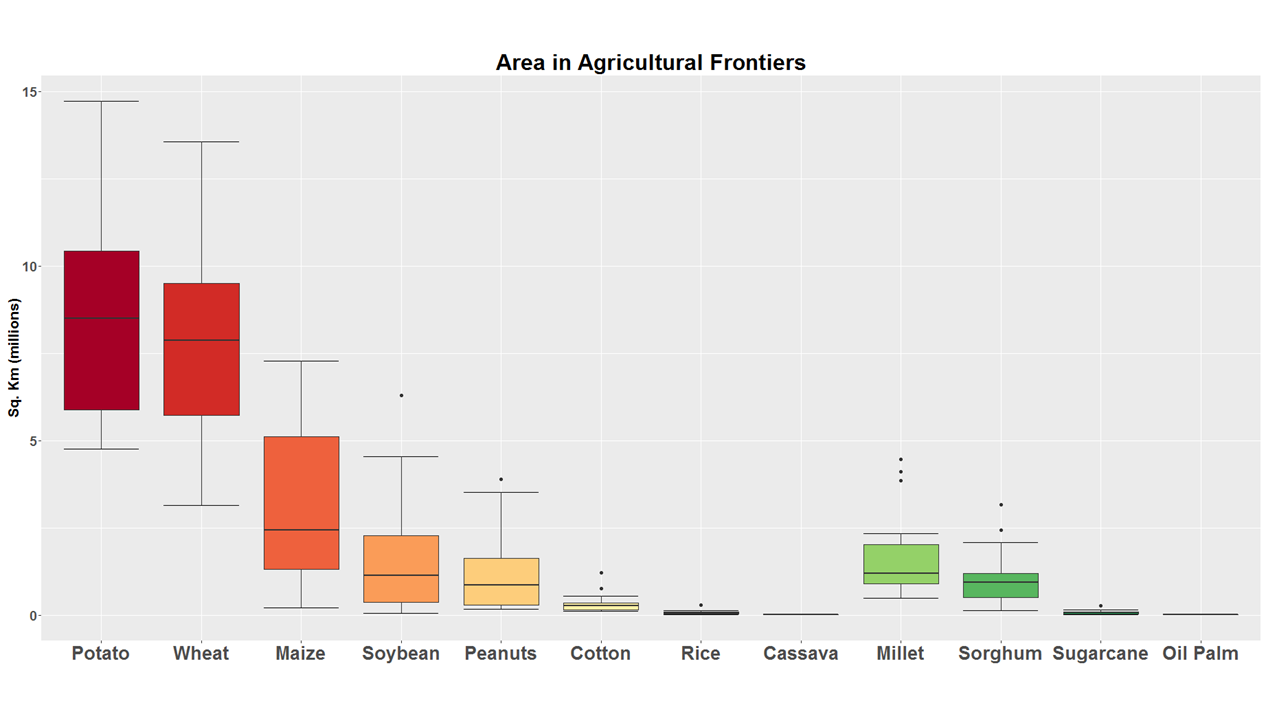

Supplement: S4 Fig — Boxes are interquartile range; whiskers extend to 1.5 times interquartile range; outliers indicated by points. (TIF) [file pone.0228305.s012.tif]

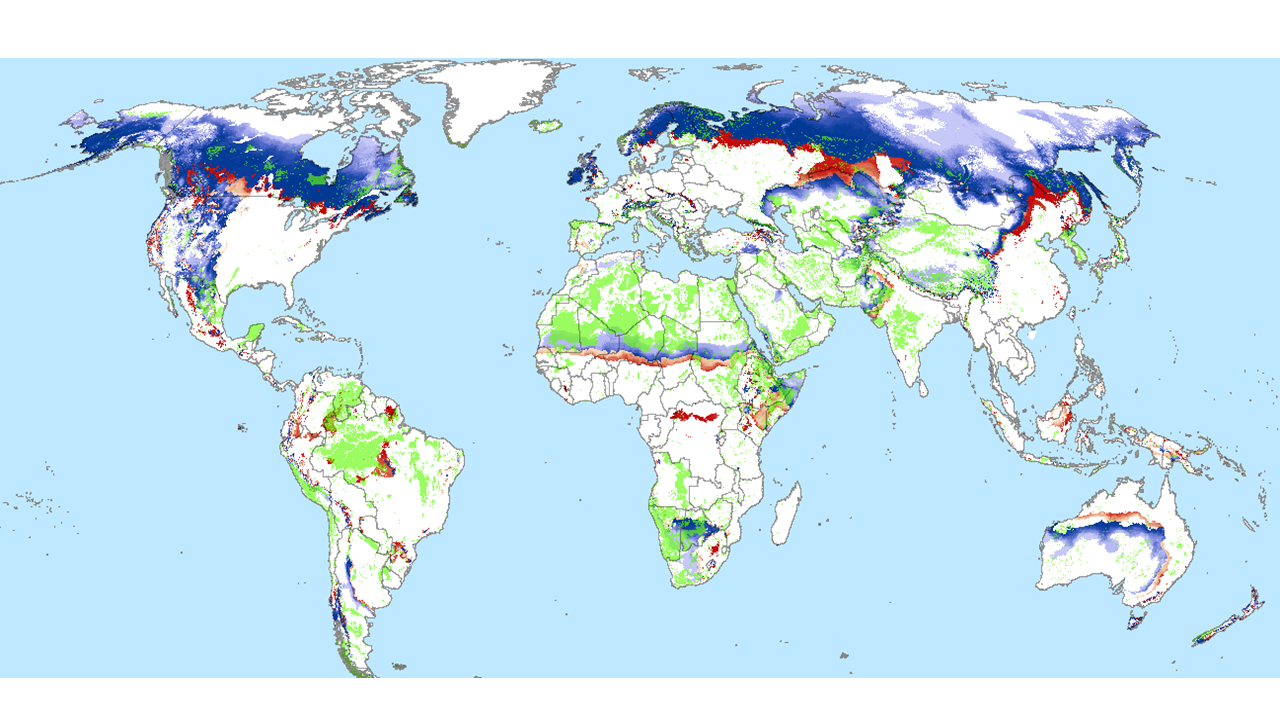

Supplement: S5 Fig — Soil constraints were defined as the union of GAEZ soil resource classifications and crop specific limitations of pH and soil depth. Areas in green were used to reduce total frontier impact on soil carbon in Table 2. (TIF) [file pone.0228305.s013.tif]

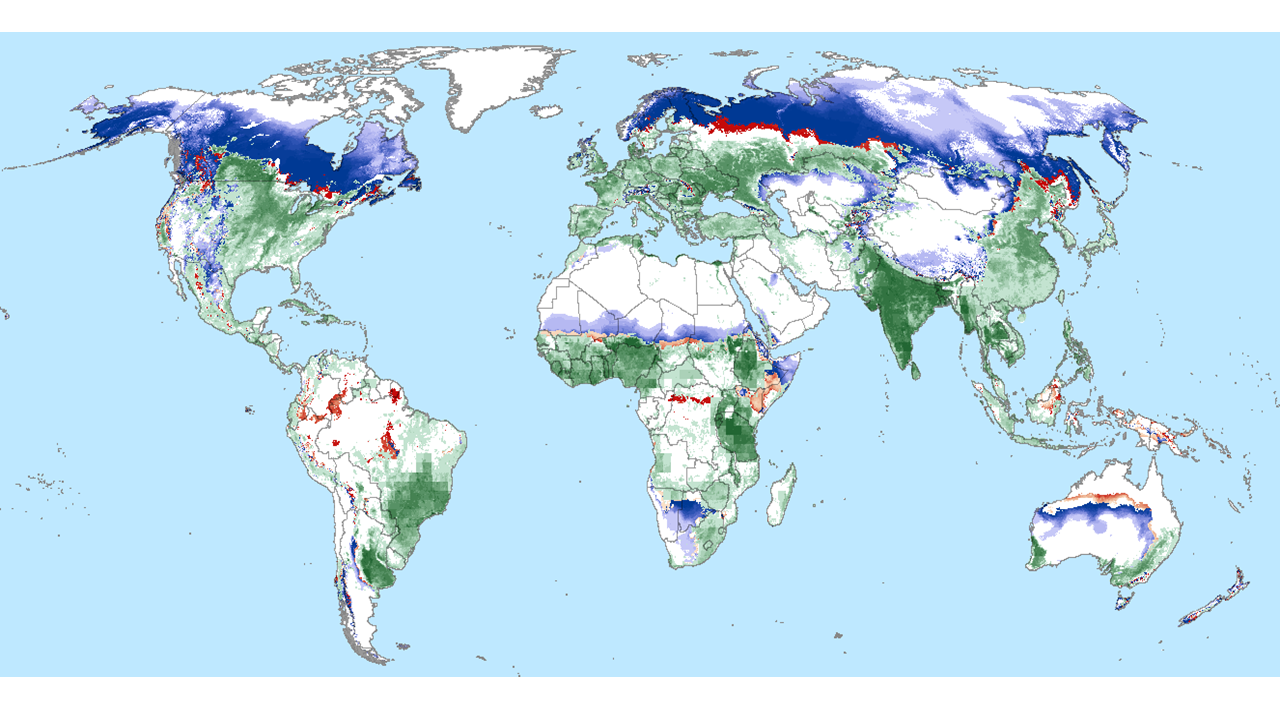

Supplement: S6 Fig — (TIF) [file pone.0228305.s014.tif]

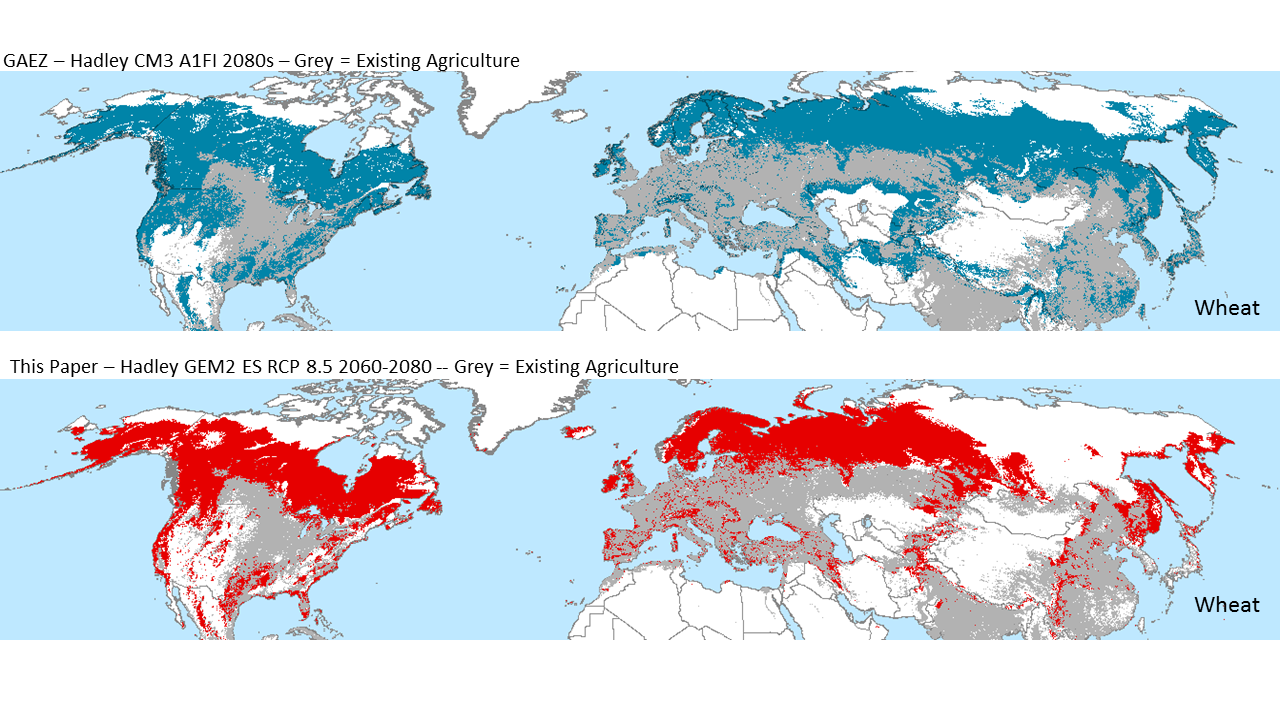

Supplement: S7 Fig — An exact match for GCM, scenario and time step was not available, but shows general spatial congruence of the modeling methods for late-century, high emissions scenarios. Existing agriculture is represented in grey for both panels. (TIF) [file pone.0228305.s015.tif]

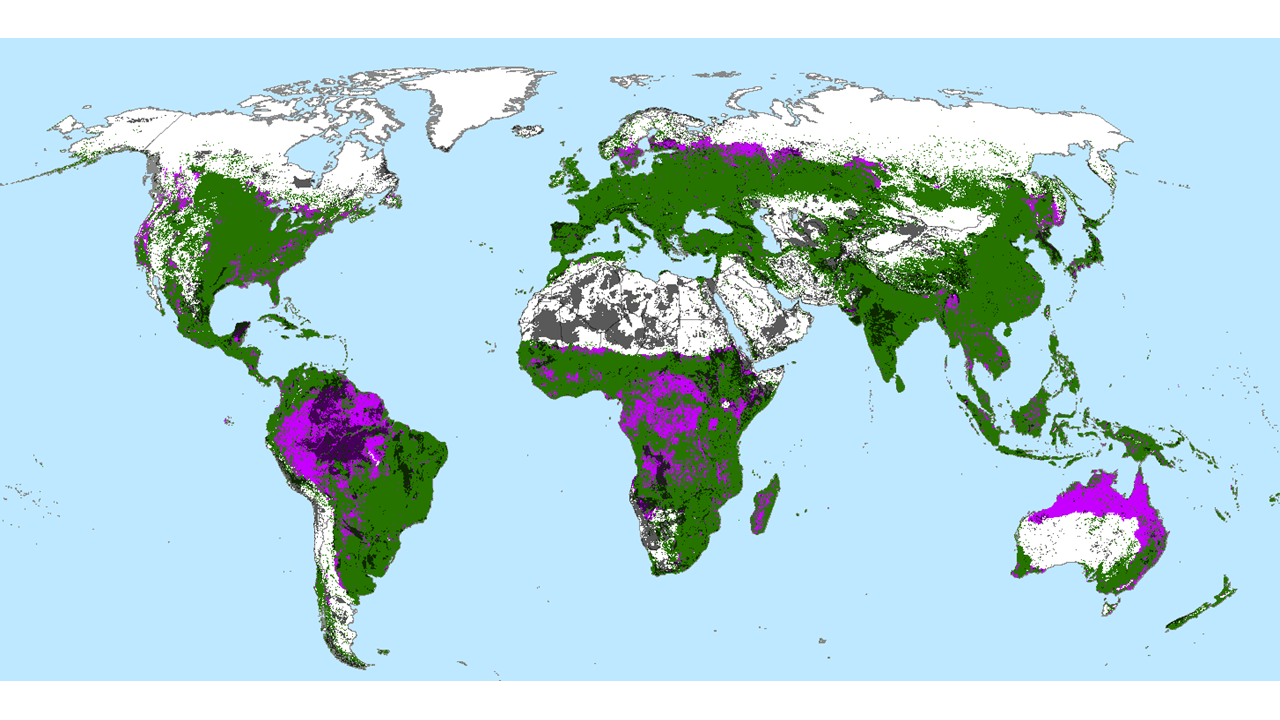

Supplement: S8 Fig — Areas of visible purple are where the universal agreement among three models indicates that it is currently suitable for at least one modeled commodity crop but is not currently cultivated. The pole ward edges of the extent of modeled vs. realized agriculture are in good agreement, with modeled agriculture extending further than existing continuous agriculture. Quantification of frontiers and their potential impacts presented here are therefore conservative. (TIF) [file pone.0228305.s016.tif]
