# Supplementary material for: The environmental consequences of climate-driven agricultural frontiers
Source: PLoS One. 2020 Feb 12;15(2):e0228305. doi: 10.1371/journal.pone.0228305 (PMC7015311; doi:10.1371/journal.pone.0228305)

**Table S3. List of Crops modeled and EcoCrop Parameters used.** KTmp = Killing Temperature (°C); Tn = Absolute Minimum Temp (°C) ; TnOp = Minimum Optimal Temp (°C); Tx = Absolute Maximum Temp (°C); TxOp = Maximum Optimal Temp (°C); Pn = Absolute Minimum Precip (mm) ; PnOp = Minimum Optimal Precip (mm); Px = Absolute Maximum Precip (mm); PxOp = Maximum Optimal Precip (mm); Gseas = Growing Season Duration (days)


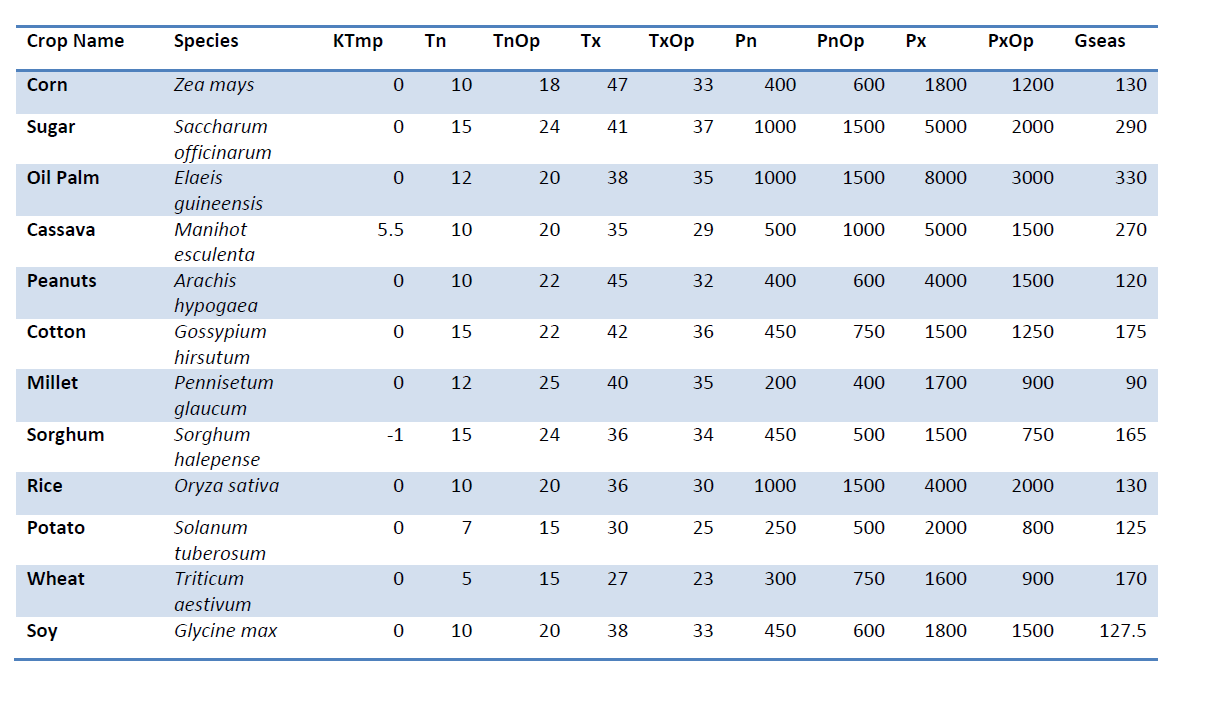

Supplement: S3 Table — KTmp = Killing Temperature (°C); Tn = Absolute Minimum Temp (°C); TnOp = Minimum Optimal Temp (°C); Tx = Absolute Maximum Temp (°C); TxOp = Maximum Optimal Temp (°C); Pn = Absolute Minimum Precip (mm); PnOp = Minimum Optimal Precip (mm); Px = Absolute Maximum Precip (mm); PxOp = Maximum Optimal Precip (mm); Gseas = Growing Season Duration (days). (DOCX) [file pone.0228305.s003.docx]
