# Supplementary material for: The environmental consequences of climate-driven agricultural frontiers
Source: PLoS One. 2020 Feb 12;15(2):e0228305. doi: 10.1371/journal.pone.0228305 (PMC7015311; doi:10.1371/journal.pone.0228305)

**Table S6**. **Summary of model performance (AUC), logistic threshold used to create binary distributions (Threshold) and the four most important variables for each crop (Var 1-4).**


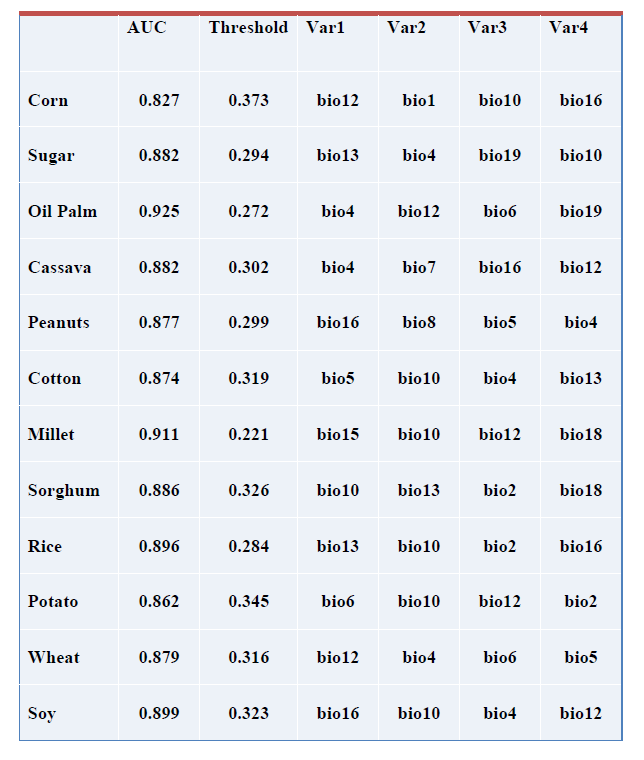

Supplement: S6 Table — (DOCX) [file pone.0228305.s006.docx]
