# Supplementary material for: The environmental consequences of climate-driven agricultural frontiers
Source: PLoS One. 2020 Feb 12;15(2):e0228305. doi: 10.1371/journal.pone.0228305 (PMC7015311; doi:10.1371/journal.pone.0228305)
Supplement: S7 Table — Critical temperature thresholds used are in bold. Critical temperature values were obtained from references [76–90]. (DOCX) [file pone.0228305.s007.docx]

**Table S7**. **Critical Maximum and Minimum temperatures for each crop.** Critical temperature thresholds used are in bold. Critical temperature values were obtained from references [78-91].
