# Supplementary material for: The environmental consequences of climate-driven agricultural frontiers
Source: PLoS One. 2020 Feb 12;15(2):e0228305. doi: 10.1371/journal.pone.0228305 (PMC7015311; doi:10.1371/journal.pone.0228305)
Supplement: S8 Table — Dice-Sorenson measures the spatial overlap with the formula 2a/2a+b+c where a = cells in common and b + c = cells in disagreement. (DOCX) [file pone.0228305.s008.docx]

**Table S8. Dice-Sorenson spatial congruence for ecocrop, maxent, and combined model with extreme temperature masks. Dice-Sorenson measures the spatial overlap with the formula 2a/2a+b+c where a = cells in common and b + c = cells in disagreement.**

| CROP | DS ECOCROP | DS MAXENT | DS COMBINED |
| --- | --- | --- | --- |
| CASSAVA | 0.38 | 0.44 | 0.48 |
| CORN | 0.49 | 0.53 | 0.54 |
| COTTON | 0.31 | 0.38 | 0.41 |
| MILLET | 0.17 | 0.40 | 0.42 |
| OILPALM | 0.06 | 0.18 | 0.03 |
| PEANUT | 0.22 | 0.34 | 0.33 |
| POTATO | 0.34 | 0.61 | 0.62 |
| RICE | 0.33 | 0.49 | 0.51 |
| SORGHUM | 0.31 | 0.42 | 0.43 |
| SOY | 0.22 | 0.44 | 0.44 |
| SUGAR | 0.23 | 0.26 | 0.27 |
| WHEAT | 0.39 | 0.63 | 0.61 |
| ALLCROPS | **0.80** | **0.81** | **0.82** |
